# Sampling Strategy and Potential Utility of Indels for DNA Barcoding of Closely Related Plant Species: A Case Study in *Taxus*

**DOI:** 10.3390/ijms13078740

**Published:** 2012-07-13

**Authors:** Jie Liu, Jim Provan, Lian-Ming Gao, De-Zhu Li

**Affiliations:** 1 Key Laboratory of Biodiversity and Biogeography, Kunming Institute of Botany, Chinese Academy of Sciences, Kunming 650201, China; E-Mail: liujie@mail.kib.ac.cn; 2 Plant Germplasm and Genomics Center, Germplasm Bank of Wild Species, Kunming Institute of Botany, Chinese Academy of Sciences, Kunming 650201, China; 3 School of Biological Sciences, Queen’s University Belfast, 97 Lisburn Road, Belfast, BT9 7BL, UK; E-Mail: j.provan@qub.ac.uk

**Keywords:** DNA barcoding, indel (gap) coding, sampling strategy, noncoding chloroplast regions, *Taxus*

## Abstract

Although DNA barcoding has become a useful tool for species identification and biodiversity surveys in plant sciences, there remains little consensus concerning appropriate sampling strategies and the treatment of indels. To address these two issues, we sampled 39 populations for nine *Taxus* species across their entire ranges, with two to three individuals per population randomly sampled. We sequenced one core DNA barcode (*mat*K) and three supplementary regions (*trn*H-*psb*A, *trn*L-*trn*F and ITS) for all samples to test the effects of sampling design and the utility of indels. Our results suggested that increasing sampling within-population did not change the clustering of individuals, and that meant within-population *P*-distances were zero for most populations in all regions. Based on the markers tested here, comparison of methods either including or excluding indels indicated that discrimination and nodal support of monophyletic groups were significantly increased when indels were included. Thus we concluded that one individual per population was adequate to represent the within-population variation in these species for DNA barcoding, and that intra-specific sampling was best focused on representing the entire ranges of certain taxa. We also found that indels occurring in the chloroplast *trn*L*-trn*F and *trn*H-*psb*A regions were informative to differentiate among for closely related taxa barcoding, and we proposed that indel-coding methods should be considered for use in future for closed related plant species DNA barcoding projects on or below generic level.

## 1. Introduction

DNA barcoding is a technique to identify species by using standardized DNA sequences [1]. It is regarded as a complementary tool to conventional taxonomic methods, and has been widely applied in fields including biodiversity inventory [2], forensic analyses [3], community phylogeny [4] and diet analysis [5]. The mitochondrial cytochrome oxidase subunit I gene (COI) is a widely used barcoding region in a range of animal groups, but is unsuitable for plant barcoding due to its low substitution rate and frequent intra-molecular recombination of the mitochondrial genome in land plants [6–8]. Although many studies have already compared the performance of a range of candidate DNA loci as barcodes in different plant taxa (e.g., [6,7,9]), the optimal choice of DNA regions adopted for plant barcoding has not yet achieved consensus [8,10,11]. Several noncoding chloroplast regions, such as *trn*H-*psb*A [6,12] and *trn*L-*trn*F [13], were proposed as DNA barcodes. Based on a comprehensive evaluation of seven candidate DNA regions on a large dataset, the Consortium for the Barcode of Life (CBOL) Plant Working Group [11] recommended the two-marker combination of the chloroplast *mat*K + *rbc*L genes as the core barcode for land plants, and suggested *trn*H*-psb*A and ITS as supplementary DNA barcodes. Recently, the China Plant BOL Group has also suggested that the nuclear internal transcribed spacer (ITS) of the ribosomal DNA should be incorporated into the core barcode for seed plants together with *mat*K and *rbc*L [9].

Although many studies on barcoding in plants have been carried out to identify useful barcoding markers [6,7,9,11,14] and to test/apply these markers in selected groups of interest (e.g., [15–17]), depth of intraspecific sampling in such studies is usually sacrificed in favor of greater taxonomic coverage [18,19]. This is a potentially important aspect of DNA barcoding, since insufficient taxon sampling may hinder the accurate assignment of query sequences with distance-based methods due to incomplete or geographically restricted sampling [19,20]. Conversely, excessive sampling for DNA barcoding may result in a waste of effort and increased cost. Thus, the appropriate number of individuals per population and populations per species required for reliable plant DNA barcoding needs to be investigated further. Although sample sizes of 5–10 specimens per species are suggested in the DNA barcoding database (http://www.barcodinglife.org/views/login.php), how good a representation this is of intraspecific variation remains unclear [21].

Indel characters have been shown to be phylogenetically informative (e.g., [22–24]), with the potential to increase the resolution of evolutionary relationships among taxa [23]. Among plant candidate DNA barcoding regions, non-coding regions, such as the chloroplast markers *trn*H-*psb*A and *trn*L*-trn*F, and the nuclear ITS usually exhibit high levels of variation, including indel polymorphism [25], and provide good capacity for species identification [6,8,13]. Although indels are often considered as a severe limitation in using such DNA regions for plant barcoding at higher taxonomic levels [11], they can be potentially useful for discriminating between closely related taxa [12]. However, very few studies have evaluated the extent of indel utilization in DNA barcoding (e.g., [26,27]). Different treatments of indels have been performed in plant DNA barcoding studies, from treating them as missing data [14], as a fifth character [28], and various methods of indel coding [26] to complete removal. As the proposed DNA barcoding regions for land plants generally exhibit relatively low levels of sequence variation, it is important to assess how to use the information associated with indels when attempting to discriminate between closely related or relatively recently evolved species [28].

In a previous study of DNA barcoding for Eurasian *Taxus* species based on five DNA regions (*rbc*L, *mat*K, *trn*L*-trn*F, *trn*H-*psb*A and ITS), eleven species were clearly identified [29]. Among them, seven species (*T. baccata*, *T. fauna*, *T. wallichiana*, *T. chinensis*, *T. mairei*, *T. cuspidata* and *T. sumatrana*) corresponded to known extant species, whilst the other four indicated unnamed cryptic species (Qinling type, Emei type, Hengduan type and Tonkin type), which were confirmed by morphological evidence [30]. Despite being closely related, all the species possessed clearly defined distribution ranges and are treated as separate species in this study. The *trn*L-*trn*F region was identified as a good candidate DNA barcode for delimitation of these *Taxus* species, whilst the *trn*H-*psb*A region exhibited a relative low species identification success. However, both regions contained many indels, and thus in the present study we attempted to use *Taxus* as a model to test sampling strategy and indel treatment methods in plant DNA barcoding for closely related taxa based on an enlarged sampling at population level. The main aims of the present study were: (1) to identify optimal sample sizes of population/species in DNA barcoding of *Taxus*; and (2) to compare whether different treatments of indels could improve species identification success for closely related taxa of *Taxus*. We hope that the findings here can provide some useful guidelines for other similar plant barcoding studies.

## 2. Materials and Methods

### 2.1. Sampling Strategy

To evaluate the effect of intraspecific sampling size on DNA barcoding, two to three individuals per population, and three to eight populations for each species were collected representing its entire distribution range. In total, 103 individuals from 39 populations of nine species of Eurasian yews identified in Liu *et al.* [29] (with the exception of *T. sumatrana* and the Emei type, for which no population samples were available.), were used in the present study (Supplementary Information, Table S1). Among the 103 individuals, 156 sequences from 39 individuals were based on Liu *et al.* [29], and 256 sequences from 64 individuals were newly sequenced in the present study (Supplementary Information, Table S1). Voucher specimens were deposited in the herbaria of Kunming Institute of Botany (KUN) and/or Royal Botanic Garden Edinburgh (E).

### 2.2. DNA Extraction, PCR and Sequencing

Total genomic DNA was isolated using a modified 4 × CTAB method [31] from silica-gel dried leaf materials. PCR amplification and sequencing of the *mat*K, *trn*L*-trn*F, *trn*H-*psb*A and ITS regions were performed as described by Liu *et al.* [29].

### 2.3. Data Analysis

Sequences were assembled and edited using SeqMan (DNA STAR package, DNAStar Inc., Madison, WI, USA). Each DNA region was aligned using CLUSTAL × 2.0 with default parameters [32], and the alignment modified manually where required under EditPlus Text Editor 3.20 [33]. Sequences were checked against those in GenBank using the BLAST algorithm. All sequences have been submitted to GenBank (Supplementary Information, Table S1).

For the sampling strategy, we used three criteria to identify the optimal intraspecific sampling. The first was the genetic distance at the population and species level; the second was the haplotype diversity of given species, and the last was clustering of the accessions using a tree building approach. Inter- and intra-specific *P*-distances for each locus were calculated using MEGA 4.0 [34] at both the population and species levels. Populations WX and KV were excluded from the intra-population analyses due to only a single individual being sequenced successfully. The number of haplotypes per locus per species was determined using MEGA 4.0. The neighbor-joining (NJ) trees were constructed with the *P*-distance model in MEGA 4.0. Bootstrap value for all clades was assessed with 5000 bootstrap replicates. The optimal sampling strategy for DNA barcoding was identified as the point where increasing the numbers of individuals at the population or species level did not change further the genetic distance, and clustering relationships of the given species. Of course, we also considered the number of haplotypes found in each species when we defined the idea sampling strategy.

Alignment of the two non-coding chloroplast regions (*trn*L*-trn*F and *trn*H-*psb*A) revealed the occurrence of many indels. Thus, we used these two data sets to test the utility of the indels for barcoding closely related species by using four different indel treatments, (1) complete deletion (abbreviated hereafter as CD), sites containing alignment were removed prior to the analysis; (2) pairwise deletion (PWD), in which indels were removed during the analysis as the need arises (e.g., pairwise distance computation); (3) simple indel coding (SIC), which was implemented by coding all indels that have different 5′ and/or 3′ termini as separate presence/absence characters; in this method, whenever a gap from one sequence contains a smaller gap in another sequence, the longer, completely overlapping gap is coded as inapplicable (Figure 1 in [22]); (4) modified complex indel coding (MCIC), which not only corrected costs downwards compared to the complex indel coding method by Simmons and Ochoterena [22], but also maintained symmetry for all step matrices, and treated overlapping indels as multistate characters [35]. To compare these four indel treating schemes, we used a tree-building method to identify the species identification success by assessing bootstrap values as described by Liu *et al*. [29]. If all of the individuals of the given species were clustered in a clade with a nodal support value of greater than 50%, we considered this as successful sequence identification. NJ trees were constructed using the *P*-distance model in MEGA 4.0 as described above. Numbers of variable sites and parsimony-informative sites for each region were also estimated with MEGA 4.0.

## 3. Results

### 3.1. Sequence Characters of the Four Loci

The *mat*K alignment was 1533 bp in length, with no length variation in any of the sampled individuals (Table 1). Length variation was observed in the *trn*L*-trn*F, *trn*H-*psb*A and ITS sequences (Table 1), which ranged from 797 bp to 852 bp, 532 bp to 981 bp and 1135 bp to 1141 bp, respectively. The aligned matrix of *trn*L*-trn*F was 869 characters in length with 11 indels which ranged from 1 to 41 bp. The length of *trn*H-*psb*A matrix had 1321 characters including 13 indels, ranging from 1 to 474 bp in length. The length of ITS aligned matrix was 1143 with 5 mononucleotide indels.

### 3.2. Genetic Distance and Clustering Relationship

Mean interspecific and intraspecific *P*-distances differed among the four regions (Table 1). Seven of the nine species exhibited no intra-specific variation for *mat*K, whilst four and five species lacked intra-specific variation for both the *trn*L*-trn*F and *trn*H-*psb*A regions (Table 2). For ITS, three species showed no intra-specific variation (Table 2). One to three haplotypes per species were found in the *mat*K region, and one to eight, one to ten and one to six were found for *trn*L-*trn*F, *trn*H-*psb*A and ITS, respectively (Table 2). Among the 37 multiple-sampled populations of the nine species, only one population of *T. fuana* (GL) exhibited polymorphism for *mat*K, eight populations were polymorphic for *trn*H-*psb*A, and eleven for both *trn*L*-trn*F and ITS (Supplementary Information, Table S2). For the polymorphic populations within each species for the four regions, intra-population *P*-distances were generally less than inter-population and intra-specific *P*-distances (Table 2).

**Figure 1:**
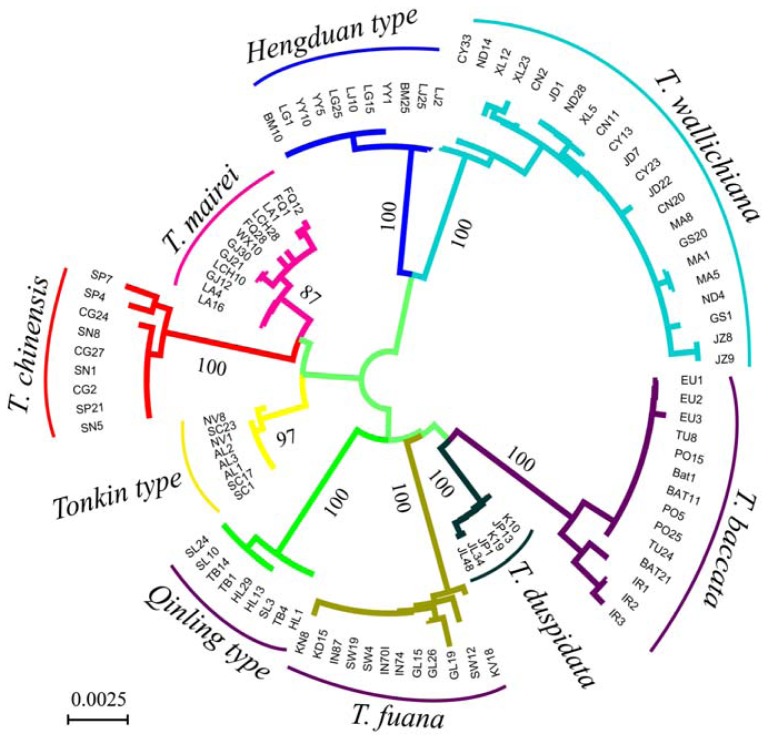
Unrooted neighbour-joining (NJ) tree based on the *P*-distance of the five DNA barcoding loci used. Bootstrap values are shown along the branch for each clade. Scale bar represents base substitutions per site.

The NJ tree of the nine species based on combination of the four DNA regions is shown in Figure 1. All the 103 individuals fell into distinct clades with high bootstrap support values corresponding to the nine species. The clustering relationships of the species were similar to those found by Liu *et al*. [29], which utilized smaller sample sizes for each species.

### 3.3. Indel-Treating Method Comparison

The numbers of variable and parsimony-informative characters varied between the two indel coding approaches (SIC and MCIC) for *trn*L*-trn*F and *trn*H-*psb*A (Table 1). The variable sites and parsimony-informative characters ranged from 2.88% to 4.09% with MCIC, and 2.53% to 3.30% with SIC for *trn*L*-trn*F, and ranged from 1.29% to 2.75% and 0.98% to 1.97% with MCIC and SIC for *trn*H-*psb*A, respectively (Table 1). When comparing numbers of the variable sites and parsimony-informative characters, CD = PWD < MCIC < SIC. Indel-coding increased the numbers of variable and parsimony-informative characters, and SIC generally resulted in more variable and parsimony-informative sites than MCIC (Table 1).

NJ trees for each region were constructed with different indel treatments by MEGA 4.0. Clades were recovered for each species and nodal support values are shown in Table 3. Nodal support values base on SIC and MCIC were always higher than those using CD and PWD in the *trn*L*-trn*F NJ tree, except for *T. cuspidata* which had the highest bootstrap support with PWD. All the nodal support values for each clade were over 70% except for a value of 58% in the Qinling type. Seven out of the nine species were identified with CD and PWD, eight with SIC, and all nine species with MCIC. For *trn*H-*psb*A, PWD, SIC, MCIC all performed better than CD on species resolution and nodal support. Only one of the nine species was discriminated with CD, and three with PWD. SIC and MCIC provided matching species discriminatory power, identifying four of the nine species (44%) (Table 3). It is of note that two and one of the nine species could be distinguished using the *trn*L-*trn*F and *trn*H*-psb*A regions respectively when considering only indels variation (Table 3).

## 4. Discussion

### 4.1. Sampling Size of Population/Species for Plant Barcoding

The number of individuals required to create a reliable reference for valid species identification has been one of the basic issues considered since the beginning of the DNA barcoding initiative [18,21]. Generally, the depth of individuals per species sampled for barcoding is usually sacrificed in favor of greater taxonomic coverage [36]. In the present study, levels of sequence divergence within and between species differed among the four DNA regions in this study, most likely as a result of different evolutionary processes/levels of functional constraint. Little or no intraspecific sequence divergence was observed in *mat*K, indicating that 2–3 individuals from different populations would be representative of the genetic diversity of each species (Table 2). For more quickly evolving regions, such as *trn*H*-psb*A, *trn*L-*trn*F and ITS, high levels of intraspecific sequence divergence mean that more individuals (a maximum of 10, 8 and 6) from different populations are required to represent the majority of the genetic variation at these loci (Table 2). Although a sample size of 12 individuals per species was proposed for barcoding animals by Matz & Nielsen [18], a study based on simulated data and real data from the *mt*DNA COI region for the skipper butterfly [21] suggested that much larger sample sizes were required to be representative of the total genetic diversity. Based on the results in this study and those from a separate phylogeographic study of *Taxus* (Liu *et al.*, unpublished data), 8–10 individuals per species from the entire geographic distribution of the species analysed appear to be sufficient for plant DNA barcoding. Nevertheless, more detailed studies on this issue are required using simulated data and real data on a large sample scale in future.

In our study, intraspecific sampling was conducted in a hierarchical fashion to elucidate whether variation between populations could influence sampling strategies for barcoding. Most of the populations studied for multiple individuals were monomorphic for all four DNA regions (Supplementary Information, Table S2). Intra-population sequence divergence was usually less than that observed between populations within species (Table 2), and mean interspecific distances (Table 1) and clustering relationships of the species in the NJ tree (Figure 1) were similar to a previous study which only considered a single individual per population [29]. Thus, increasing numbers of individuals within populations does not influence barcoding accuracy, and one individual per population is likely to be adequate for the majority of plant DNA barcoding projects, especially for the closely related taxa. Most of the variation within species is generally due to differences between geographically distant populations. Thus it is more important to randomly sample multiple individuals across the whole geographical distribution of a species, a similar finding to that from a previous study [21].

### 4.2. Utility of Indels for Barcoding and Effect of Different Indel Treatments

As methods for coding indels have become more sophisticated, the inclusion of indels as characters in phylogenetic analyses has gained increasing popularity [37]. Many studies concluded that gaps should be included, in some way, in order to provide additional phylogenetic information (e.g., [23,24,28]). In plant DNA barcoding studies, indels have generally not been taken into account due to the lack of a standard approach to the utilization of such regions. Studies have generally treated indels as missing data [6,11,12], in a few cases, as a fifth character [28] or as some indel coding method [26].

Length variation as a result of mononucleotide repeat expansion and contraction in the chloroplast genome has frequently been utilized as a marker system in population genetic studies [38]. The bidirectional nature of the mutational processes operating at these regions, however, leads to homoplasy, particularly above the species level, and thus they are not generally considered as informative in phylogenetic studies, or even in phylogeographic studies across large geographic scales, e.g., [39]. In the present study, as in previous other studies, e.g., [40], similarly, polymorphic mononucleotide regions have been identified in the nuclear ITS. Given that these result from the same mutational mechanisms as their chloroplast counterparts, they are unlikely to provide stable information for inter-specific barcoding studies.

Rapidly evolving non-coding plant DNA regions, such as *trn*H-*psb*A and *trn*L*-trn*F, usually exhibit length variation due to the occurrence of indels, e.g., [7,41]. Where such length variation makes it difficult to unambiguously align sequences, this represents a disadvantage in DNA barcoding [11], although this is primarily a problem in distantly related taxa. Conversely, the potentially diagnostic nature of indels for closely related species is highlighted in the present study, as well as in previous barcoding researches [6,12,42]. Our study shows that some species have private intraspecific indels that distinguish them from other closely related species. For instance, *T. chinensis* has a specific insertion at 514 position in *trn*H-*psb*A matrix which differentiated it from the other species studied. Likewise, *T. mairei* can only be distinguished from the ‘Tonkin type’ by a private indel in the *trn*L*-trn*F region.

Given that indels are known to provide some level of information for phylogenetic studies, e.g., [24,37], we utilised four different methods of indel treatment to test their performance in DNA barcoding analysis. Based on our results, the two gap coding methods (SIC or MCIC) performed better in species discrimination and nodal support than CD and PWD, and both approaches usually obtained similar species resolution and nodal support values. A comparison of the CD and PWD approaches revealed that they performed similarly in *trn*L*-trn*F, but that PWD performed better in species identification than CD in *trn*H-*psb*A. Overall, the CD method showed the lowest species discrimination and nodal support. Using this approach, where indels were effectively treated as missing data, the resulting phylogentic trees were less accurate than those obtained using gap coding treatments [24]. In general, gap coding methods of SIC and MCIC increased the number of variable and parsimony-informative sites, which gave more accurate clustering relationships and a stronger nodal support value. Where the indel was a species-specific diagnostic character, this always led to the accessions of the species forming a clade and subsequent species identification success. Thus, for the barcoding of the closely related species of *Taxus* examined in this study, it was preferable to use a gap-coding method (SIC or MCIC) rather than the CD approach.

The findings of the study confirm the potential utility of indels in plant barcoding studies, as previously suggested in other species [42]. Considering that the rate of base pair substitutions in the chloroplast genome is fairly low [43,44], longer stretches of DNA may be required to generate sufficient barcoding information, which will in turn increase the cost of such ventures. Given the high frequency of indels, particularly in the two regions studied here, and their phylogenetic utility in species discrimination, we suggest that with the appropriate treatment, they may provide a valuable addition to many plant barcoding studies.

## Supplementary Information



## Figures and Tables

**Table 1:** Summary of data sets by indel coding scheme for alignment and analyses.

Data set	Indel treating method	No. of indel	Aligned length	No. (%) VC	No. (%) PIC	Mean interspecific distance	Mean intraspecific distance
*mat*K	-	0	1533	15 (0.98)	15 (0.98)	0.0027 (0.00065–0.0046)	0.00006 (0–0.00024)
*trn*L-*trn*F	-	11	869	25 (2.88)	22 (2.53)	0.0063 (0.00083–0.0063)	0.00034 (0–0.0011)
	SIC		880	36 (4.09)	29 (3.30)	-	
	MCIC		874	30 (3.43)	26 (2.98)	-	
*trn*H-*psb*A	-	13	1321	17 (1.29)	13 (0.98)	0.0075 (0–0.013)	0.00046 (0–0.0014)
	SIC		1334	30 (2.25)	22 (1.65)	-	
	MCIC		1330	26 (1.96)	21 (1.57)	-	
ITS	-	5	1143	51 (4.46)	44 (3.85)	0.010 (0.0046–0.015)	0.00042 (0–0.00096)

Notes: Char, character; PIC, parsimony-informative character; VC, variable character; SIC, simple indel coding; MCIC, modified complex indel coding. All data sets have 103 taxa.

**Table 2:** Estimates of average evolutionary divergence over sequence pairs within population and between population levels, and mean intraspecific distance.

		*mat*K				*trn*L-*trn*F				*trn*H-*psb*A				ITS			
		
Lineage	*N*	*N*_H_	Within population distance	Between population distance	Intraspecific distance	*N*_H_	Within population distance	Between population distance	Intraspecific distance	*N*_H_	Within population distance	Between population distance	Intraspecific distance	*N*_H_	Within population distance	Between population distance	Intraspecific distance
Hengduan type	11	1	0	0	0	1	0	0	0	1	0	0	0	2	0–0.00088	0–0.00088	0.00038
Qinling type	9	1	0	0	0	1	0	0	0	3	0–0.00188	0–0.00188	0.00094	1	0	0	0
*Taxus baccata*	14	3	0	0–0.00067	0.00024	4	0–0.00124	0–0.00369	0.00095	2	0	0–0.0387	0.0014	2	0–0.00351	0–0.00357	0.00050
*T. chinensis*	9	1	0	0	0	1	0	0	0	1	0	0	0	4	0–0.00527	0–0.00527	0.0019
*T. cuspidata*	6	1	0	0	0	1	0	0	0	1	0	0	0	2	0	0–0.00088	0.00047
*T. fuana*	12	3	0–0.00065	0–0.00130	0.00031	2	0–0.00249	0–0.00249	0.00041	3	0–0.00376	0–0.00564	0.00094	1	0	0	0
*T. mairei*	12	1	0	0	0	3	0–0.00125	0–0.00125	0.00060	1	0	0	0	6	0–0.00264	0–0.00264	0.00096
*T. wallichiana*	22	1	0	0	0	8	0–0.00124	0–0.00369	0.0011	10	0–0.00362	0–0.00519	0.00084	3	0–0.00176	0–0.00176	0.00026
Tonkin type	8	1	0	0	0	2	0	0	0	1	0	0	0	1	0	0	0

Notes: *N*, number of individuals; *N*_H_, number of haplotypes.

**Table 3:** Bootstrap values (%) of different lineages based on different indel coding approaches conducted in MEGA 4.0.

Region	Indel coding schemes	Hengduan type	Qinling type	*T. baccata*	*T. chinensis*	*T. cuspidata*	*T. fuana*	*T. mairei*	*T. wallichiana*	Tonkin type	Resolution (%)
*trn*L-*trn*F	CD	77	n.d.	97	83	76	76	n.d.	87	77	77.8 (7/9)
	PWD	80	n.d.	98	84	90	79	n.d.	86	73	77.8 (7/9)
	SIC	79	58	98	94	74	84	n.d.	91	81	88.9 (8/9)
	MCIC	72	77	99	97	80	86	40	91	89	100 (9/9)
*trn*H-*psb*A	CD	n.d.	n.d.	n.d.	n.d.	62	n.d.	n.d.	n.d.	n.d.	11.1 (1/9)
	PWD	n.d.	n.d.	n.d.	64	95	n.d.	n.d.	93	n.d.	33.3 (3/9)
	SIC	58	n.d.	n.d.	64	96	n.d.	n.d.	93	n.d.	44.4 (4/9)
	MCIC	55	n.d.	n.d.	64	96	n.d.	n.d.	98	n.d.	44.4 (4/9)

CD, complete deletion; PWD, pairwise deletion. Note: n.d., “taxa” not distinguished.
