# Supplementary material for: Sampling Strategy and Potential Utility of Indels for DNA Barcoding of Closely Related Plant Species: A Case Study in Taxus
Source: Int J Mol Sci. 2012 Jul 13;13(7):8740–51. doi: 10.3390/ijms13078740 (PMC3430262; doi:10.3390/ijms13078740)
Supplement: Supplementary file 1 [file ijms-13-08740-s001.pdf]

Sampling Strategy and Potential Utility of Indels for DNA Barcoding of Closely Related Plant Species: A Case Study in *Taxus*

Supplementary Information

Table S1. List of *Taxus* samples included in this study, voucher information, GenBank accession numbers, and herbarium acronym.

Lineage	Sample code	*mat*K	*trn*L-*trn*F	*trn*H-*psb*A	ITS	Voucher specimen	Herbarium
Hengduan type	BM10	HM590987	HM591124	HM591078	HM590944	GM-24171	KUN/E
	BM25	**JX174662**	**JX188601**	**JX188478**	**JX188542**	GM-24186	KUN/E
	LG1	**JX174663**	**JX188602**	**JX188479**	**JX188543**	GM-24192	KUN/E
	LG15	HM590988	HM591125	HM591079	HM590945	GM-24201	KUN/E
	LG25	**JX174664**	**JX188603**	**JX188480**	**JX188544**	GM-24216	KUN/E
	LJ2	**JX174665**	EU052221	**JX188481**	**JX188545**	GM-2433	KUN/E
	LJ10	HM590986	HM591123	HM591077	HM590943	GM-2441	KUN/E
	LJ25	**JX174666**	**JX188604**	**JX188482**	**JX188546**	GM-2456	KUN/E
	YY1	**JX174667**	**JX188605**	**JX188483**	**JX188547**	GF-040911-29-1	KUN
	YY5	HM590989	HM591126	HM591080	HM590946	GF-040911-29-5	KUN
	YY10	**JX174668**	**JX188606**	**JX188484**	**JX188548**	GF-040911-29-10	KUN
Qinling type	HL1	**JX174669**	**JX188607**	**JX188485**	**JX188549**	LJ-05-1024	KUN/E
	HL13	HM591005	HM591142	HM591096	HM590962	LJ-05-1024	KUN/E
	HL29	**JX174670**	**JX188608**	**JX188486**	**JX188550**	LJ-05-1052	KUN/E
	SL3	**JX174672**	**JX188610**	**JX188488**	**JX188552**	LJ-05-1055	KUN/E
	SL10	HM591006	HM591143	HM591097	HM590963	LJ-05-1062	KUN/E
	SL24	**JX174671**	**JX188609**	**JX188487**	**JX188551**	LJ-05-1076	KUN/E
	TB1	**JX174673**	**JX188611**	**JX188489**	**JX188553**	MMO 05-677	KUN/E
	TB4	**JX174674**	**JX188612**	**JX188490**	**JX188554**	MMO 05-680	KUN/E
	TB14	HM591004	HM591141	HM591095	HM590961	MMO 05-694	KUN/E
Tauxs baccata	Bat1	HM591019	EF680264	HM591113	EF680244	Fior S 1	E
	Bat11	**JX174675**	**JX188613**	**JX188491**	**JX188555**	Fior S 11	E
	Bat21	**JX174676**	**JX188614**	**JX188492**	**JX188556**	Fior S 21	E
	EU1	**JX174677**	**JX188615**	**JX188493**	**JX188557**	20010206A	E
	EU2	HM591017	EF680268	HM591111	EF680248	Aberlady4	E
	EU3	**JX174678**	EF680267	**JX188494**	EF680247	AA-442-91	E
	IR1	**JX174679**	**JX188616**	**JX188495**	**JX188558**	M. Zarrei-476	E
	IR2	HM591021	HM591155	HM591115	HM590977	M. Zarrei-477	E
	IR3	**JX174680**	EF680269	**JX188496**	EF680249	M. Zarrei-478	E
	PO5	**JX174682**	EF680265	**JX188498**	EF680245	Moncao-5	E
	PO15	HM591018	HM591154	HM591112	HM590976	Moncao-15	E
	PO25	**JX174681**	**JX188617**	**JX188497**	**JX188559**	Moncao-25	E

	TU8	HM591020	EF680266	HM591114	EF680246	AAD 11812-8	E
	TU24	**JX174683**	**JX188618**	**JX188499**	**JX188560**	AAD 12043-3	E
T. chinensis	CG2	HM590991	HM591128	HM591082	HM590948	MMO 05-597	KUN/E
	CG24	**JX174684**	**JX188619**	**JX188500**	**JX188561**	MMO 05-624	KUN/E
	CG27	**JX174685**	**JX188620**	**JX188501**	**JX188562**	MMO 05-627	KUN/E
	SN1	**JX174686**	**JX188621**	**JX188502**	**JX188563**	MMO 05-488	KUN/E
	SN5	**JX174687**	**JX188622**	**JX188503**	**JX188564**	MMO 05-504	KUN/E
	SN8	HM590992	HM591129	HM591083	HM590949	MMO 05-507	KUN/E
	SP4	**JX174688**	**JX188623**	**JX188504**	**JX188565**	LJ-05-996	KUN/E
	SP7	**JX174689**	**JX188624**	**JX188505**	**JX188566**	LJ-05-999	KUN/E
	SP21	HM590990	HM591127	HM591081	HM590947	LJ-05-999	KUN/E
T. cuspidata	JL34	**JX174690**	**JX188625**	**JX188506**	**JX188567**	LJM-34	KUN/E
	JL48	HM591009	HM591147	HM591103	HM590969	LJM-48	KUN/E
	JP1	**JX174691**	**JX188626**	**JX188507**	**JX188568**		KUN
	JP13	HM591011	HM591149	HM591105	HM590971	Kokubugata-6972	KUN
	K10	**JX174692**	**JX188627**	**JX188508**	**JX188569**	WLS-10	KUN
	K19	HM591010	HM591148	HM591104	HM590970	WLS-19	KUN
T. fuana	GL15	HM591016	HM591153	HM591110	HM590975	GLM-081628	KUN
	GL19	**JX174693**	**JX188628**	**JX188509**	**JX188570**	GLM-081632	KUN
	GL26	**JX174694**	**JX188629**	**JX188510**	**JX188571**	GLM-081639	KUN
	IN70	**JX174695**	**JX188630**	**JX188511**	**JX188572**	Yuhy-70	KUN
	IN74	HM591015	HM591152	HM591109	HM590974	Yuhy-74	KUN
	IN87	**JX174696**	**JX188631**	**JX188512**	**JX188573**	Yuhy-84	KUN
	KD15	HM591014	HM591151	HM591108	HM590973	Amin-25102	KUN
	KN8	**JX174697**	**JX188632**	**JX188513**	**JX188574**	Amin-25068	KUN
	KV18	HM591012	EF680261	HM591106	EF680241	Amin-25332	KUN
	SW4	**JX174699**	**JX188633**	**JX188515**	**JX188575**	Amin-25229	KUN
	SW12	**JX174698**	EF680260	**JX188514**	EF680240	Amin-25237	KUN
	SW19	HM591013	HM591150	HM591107	HM590972	Amin-25244	KUN
T. mairei	FQ1	**JX174700**	**JX188634**	**JX188516**	**JX188576**	ZHXM0506141	KUN
	FQ12	HM591002	HM591139	HM591093	HM590959	ZHXM0506152	KUN
	FQ28	**JX174701**	**JX188635**	**JX188517**	**JX188577**	ZHXM0506168	KUN
	GJ12	HM591001	HM591138	HM591092	HM590958	GLM-05971	KUN
	GJ21	**JX174702**	**JX188636**	**JX188518**	**JX188578**	GLM 05-980	KUN
	GJ30	**JX174703**	**JX188637**	**JX188519**	**JX188579**	GLM 05-989	KUN
	LA1	HM591003	HM591140	HM591094	HM590960	ZHXM050691	KUN
	LA4	**JX174705**	EU052228	**JX188521**	**JX188581**	ZHXM050694	KUN
	LA16	**JX174704**	**JX188638**	**JX188520**	**JX188580**	ZHXM0506106	KUN
	LCH10	HM591000	HM591137	HM591091	HM590957	Zeng7-10	KUN
	LCH28	**JX174706**	**JX188639**	**JX188522**	**JX188582**	Zeng7-28	KUN
	WX10	HM590999	HM591136	HM591090	HM590956	GLM-07623	KUN
T. wallichiana	CN2	**JX174708**	**JX188641**	**JX188524**	**JX188584**	GLM-081810	KUN

	CN11	**JX174707**	**JX188640**	**JX188523**	**JX188583**	GLM-081819	KUN
	CN20	HM590984	HM591121	HM591075	HM590941	GLM-081828	KUN
	CY13	**JX174709**	**JX188642**	**JX188525**	**JX188585**	GLM-082139	KUN
	CY23	HM590978	HM591116	HM591069	HM590936	GLM-2149	KUN
	CY33	**JX174710**	**JX188643**	**JX188526**	**JX188586**	GLM-082159	KUN
	GS1	HM590982	EF680273	HM591073	EF680253	GLM-2301	KUN
	GS20	**JX174711**	EU052219	**JX188527**	**JX188587**	GLM-2334	KUN
	JD1	HM590980	HM591118	HM591071	HM590938	GM-24222	KUN
	JD7	**JX174713**	EU052218	**JX188529**	**JX188588**	GM-24228	KUN
	JD22	**JX174712**	EU052217	**JX188528**	**JX188589**	GM-24243	KUN
	JZ8	HM590981	HM591119	HM591072	HM590939	Liuj-09228	KUN
	JZ9	**JX174714**	**JX188644**	**JX188530**	**JX188590**	Liuj-09229	KUN
	MA1	HM590979	HM591117	HM591070	HM590937	GLM-06257	KUN/E
	MA5	**JX174715**	**JX188645**	**JX188531**	**JX188591**	GLM-06261	KUN/E
	MA8	**JX174716**	**JX188646**	**JX188532**	**JX188592**	GLM-06314	KUN/E
	ND4	HM590985	HM591122	HM591076	HM590942	NEP001-0364	E
	ND14	**JX174717**	EF680275	**JX188533**	EF680255	NEP014	E
	ND28	**JX174718**	**JX188647**	**JX188534**	**JX188593**	NEP028	E
	XL5	HM590983	HM591120	HM591074	HM590940	LJ-07035	KUN
	XL12	**JX174719**	**JX188648**	**JX188535**	**JX188594**	LJ-07042	KUN
	XL23	**JX174720**	**JX188649**	**JX188536**	**JX188595**	LJ-07053	KUN
Tonkin type	AL1	HM590998	HM591135	HM591087	HM590953	GLM-06001	KUN
	AL2	**JX174721**	**JX188650**	**JX188537**	**JX188596**	GLM-06002	KUN/E
	AL3	**JX174722**	**JX188651**	**JX188538**	**JX188597**	GLM-06003	KUN/E
	NV1	HM590997	HM591133	HM591089	HM590955	NVSDE82	KUN/E
	NV8	**JX174723**	**JX188652**	**JX188539**	**JX188598**	NVSDE95	KUN/E
	SC1	HM590996	HM591134	HM591088	HM590954	GLM-2357	KUN/E
	SC17	**JX174724**	**JX188653**	**JX188540**	**JX188599**	GLM-2376	KUN/E
	SC23	**JX174725**	EU052214	**JX188541**	**JX188600**	GLM-2382	KUN/E

Note, their lineages names, locality are same to Liu *et al.* [29].

Table S2. Mean within population distance for four regions studied in present work.

Population code	Regions			
	***mat*K**	***trn*L-*trn*F**	***trn*H-*psb*A**	**ITS**
LG	0.00000	0.00000	0.00000	0.00000
YY	0.00000	0.00000	0.00000	0.00000
LJ	0.00000	0.00000	0.00000	0.00059
BM	0.00000	0.00000	0.00000	0.00088
HL	0.00000	0.00000	0.00125	0.00000
SL	0.00000	0.00000	0.00125	0.00000
TB	0.00000	0.00000	0.00125	0.00000
BAT	0.00000	0.00000	0.00000	0.00000
EU	0.00000	0.00082	0.00000	0.00000

IR	0.00000	0.00082	0.00000	0.00234
PO	0.00000	0.00000	0.00000	0.00000
TU	0.00000	0.00000	0.00000	0.00000
CG	0.00000	0.00000	0.00000	0.00059
SN	0.00000	0.00000	0.00000	0.00000
SP	0.00000	0.00000	0.00000	0.00410
JL	0.00000	0.00000	0.00000	0.00000
JP	0.00000	0.00000	0.00000	0.00000
K	0.00000	0.00000	0.00000	0.00000
GL	0.00043	0.00000	0.00251	0.00000
IN	0.00000	0.00000	0.00000	0.00000
KD	0.00000	0.00000	0.00188	0.00000
SW	0.00000	0.00166	0.00000	0.00000
FQ	0.00000	0.00000	0.00000	0.00147
GJ	0.00000	0.00082	0.00000	0.00059
LA	0.00000	0.00083	0.00000	0.00000
LCH	0.00000	0.00123	0.00000	0.00088
AL	0.00000	0.00000	0.00000	0.00000
NV	0.00000	0.00000	0.00000	0.00000
SC	0.00000	0.00000	0.00000	0.00000
CN	0.00000	0.00081	0.00000	0.00029
CY	0.00000	0.00000	0.00242	0.00059
GS	0.00000	0.00000	0.00121	0.00000
JD	0.00000	0.00163	0.00000	0.00000
JZ	0.00000	0.00000	0.00000	0.00000
MA	0.00000	0.00080	0.00000	0.00000
ND	0.00000	0.00081	0.00260	0.00000
XL	0.00000	0.00082	0.00000	0.00059
WX	NC	NC	NC	NC
KV	NC	NC	NC	NC

Note: n.c., not calculated.

© 2012 by the authors; licensee MDPI, Basel, Switzerland. This article is an open access article distributed under the terms and conditions of the Creative Commons Attribution license (http://creativecommons.org/licenses/by/3.0/).
